# BCG Vaccine does not Protect Against COVID-19

**DOI:** 10.2174/1874306402014010045

**Published:** 2020-11-26

**Authors:** Mohamed F. Allam, Ghada E. Amin

**Affiliations:** 1 Department of Family Medicine, Faculty of Medicine, Ain Shams University, Cairo, Egypt

**Keywords:** Antiviral treatments, COVID-19, BCG, Vaccine, Review, Immunity

## Abstract

A recent article by Jop de Vrieze (March 23, 2020) suggested that BCG vaccine could protect against COVID-19 infections. The arguments were that several European countries, like Italy, Spain, France, and Germany, which are badly affected by COVID-19, and the USA stopped vaccination of the general population by BCG and excluded it from their routine vaccination schedule. Many people started to receive doses of BCG based on that hypothesis even before its confirmation. We think that the BCG vaccine could not protect against COVID-19 because several countries like China and Iran, which are severely affected by COVID-19, still include the BCG vaccine in its routine vaccination schedule. Other arguments include that the BCG vaccine improves cell-mediated immunity with little effect on humoral immunity; Immunity against viruses, in general, is mainly humoral.

Last year, on December 31, 2019, the Chinese health authorities declared an outbreak of novel coronavirus 2019 (COVID-19). Since then, several studies and reports have discussed the possible general and specific protective measures against COVID-19, together with potential antiviral treatments [1].

A recent article by Jop de Vrieze (March 23, 2020) suggested that the BCG vaccine could protect against COVID-19 infections. The arguments were that several European countries, like Italy, Spain, France, and Germany, which were badly affected by COVID-19, and the USA stopped vaccination of the general population by BCG and excluded it from their routine vaccination schedule [2].

It is hypothesized that the BCG vaccine could stimulate the immune system and thus, protect against COVID-19. Previous studies recommended the use of BCG locally for the treatment of urinary bladder cancer and preventing its recurrence [3].

Recently, a new study was proposed to be conducted in four countries to investigate the effectiveness of BCG against COVID-19 [2]. Many people started to receive doses of BCG based on that hypothesis even before its confirmation. We think that the BCG vaccine could not protect against COVID-19 because of the next observations:

First, China, the country where this pandemic started, still includes the BCG vaccine in its routine vaccination schedule [4].

Second, Iran, one of the most affected countries worldwide with 356,792 infected patients, as of 22 August 2020 [5], vaccinates their children with BCG with a coverage rate of 99%, and the vaccine was included in their vaccination program since its establishment [6]. Similarly, Egypt, the most affected country in Africa with 97.148 infected patients, till 22 August 2020, had BCG vaccination coverage of 95% [5, 6].

Third, Spain included the BCG vaccine in their routine vaccination program from 1924 till early 1980s, when it was excluded and hence the elderly population who are mostly affected are vaccinated. The Basque Country, located in Northern Spain, continued vaccinating its population till 2013 [7]. Basque Country is the eighth largest region in Spain, regarding the number of population. Basque Country is ranked the third most affected region in Spain with the most number of cases and deaths, after Madrid and Catalonia [8], thus continuing vaccination with BCG in Basque Country for 25-30 years after other regions stoppage of routine BCG vaccination was not protective.

Fourth, a recent cohort study of 5933 adults aged 35 to 41 years (3064 vaccinated and 2869 unvaccinated) showed no statistically significant difference in the proportion of positive COVID-19 test results in the BCG-vaccinated group vs. the unvaccinated group [9]. The results of this study agree with the official position of the World Health Organization, since 12 April 2020 till date, that there is no evidence that the BCG vaccine could be protective against COVID-19 [10].

Fifth, several reports showed that BCG vaccine improves cell-mediated immunity. Only a few studies reported little effects of the BCG vaccine on humoral immunity [11]. Immunity against viruses, in general, is humoral [12]. A recent report recommended the use of plasma of recovered COVID-19 patients to treat severe COVID-19 symptoms because of high levels of specific immunoglobulin against COVID-19 [13].

## CONCLUSION

In conclusion, further investigation is required to state whether or not BCG vaccine is related to COVID-19, and before the implementation of the BCG vaccine as a protection against COVID-19.
