# BCG Vaccine does not Protect Against COVID-19

**DOI:** 10.2174/1874306402014010078

**Published:** 2020-12-31

**Authors:** Mohamed F. Allam, Ghada E. Amin

**Affiliations:** 1 Department of Family Medicine, Faculty of Medicine, Ain Shams University, Cairo, Egypt

**Correction**

**The Open Respiratory Medicine Journal**

**BCG Vaccine does not Protect Against COVID-19**

Mohamed F. Allam^1,*^ and Ghada E. Amin^1^

**The Open Respiratory Medicine Journal, 2020, 14: 45-46 **

The following changes have been made in the article:

  ***1*-** Second author’s name has been changed as follows:

    Ghada E. Amin

